# Non-invasive assessment of phosphate metabolism and oxidative capacity in working skeletal muscle in healthy young Chinese volunteers using ^31^P Magnetic Resonance Spectroscopy

**DOI:** 10.7717/peerj.2259

**Published:** 2016-07-21

**Authors:** Ming Li, Fei Chen, Huiting Wang, Wenbo Wu, Xin Zhang, Chuanshuai Tian, Haiping Yu, Renyuan Liu, Bin Zhu, Bing Zhang, Zhenyu Dai

**Affiliations:** 1 Department of Radiology, Affiliated Drum Tower Hospital of Nanjing University Medical School, Nanjing, Jiangsu, China; 2 Department of Radiology, Affiliated Yancheng Hospital, School of Medicine, Southeast University, Yancheng, Jiangsu, China; 3 Department of Neurology, Drum Tower Hospital, Affiliated to Nanjing University Medical School, Nanjing, Jiangsu, China

**Keywords:** ^31^P magnetic resonance spectroscopy, Phosphate metabolism, Oxidative capacity

## Abstract

**Background.** Generally, males display greater strength and muscle capacity than females while performing a task. Muscle biopsy is regarded as the reference method of evaluating muscle functions; however, it is invasive and has sampling errors, and is not practical for longitudinal studies and dynamic measurement during excise. In this study, we built an in-house force control and gauge system for quantitatively applying force to quadriceps while the subjects underwent ^31^P Magnetic Resonance Spectroscopy (^31^P-MRS); our aim was to investigate if there is a sex difference of phosphate metabolite change in working muscles in young heathy Chinese volunteers.

**Methods.** Volunteers performed knee-extending excises using a force control and gauge system while lying prone in a Philips 3T Magnetic Resonance (MR) scanner. The ^31^P-MRS coil was firmly placed under the middle of the quadriceps . ^31^P-MRS measurements of inorganic phosphate (Pi), phosphocreatine (PCr) and adenosine triphosphate (ATP) were acquired from quadriceps while subjects were in a state of pre-, during- and post-exercise. The PCr, Pi, PCr/Pi, PCr/ATP, pH, work/energy cost ratio (WE), k_PCr_ and oxidative capacity were compared between males and females.

**Results.** A total of 17 volunteers underwent the study. Males: *N* = 10, *age* = 23.30 ± 1.25*years*; females: *N* = 7, age = 23.57 ± 0.79 years. In this study, males had significantly greater WE (16.33 ± 6.46 vs. 7.82 ± 2.16, *p* = 0.002) than females. Among PCr, Pi, PCr/Pi, PCr/ATP, pH, kPCr and oxidative capacity at different exercise status, only PCr/Pi (during-exercise, males = 5.630 ± 1.647, females = 4.014 ± 1.298, *p* = 0.047), PCr/ATP (during-exercise, males =1.273 ± 0.219, females = 1.523 ± 0.167, *p* = 0.025), and ATP (post-exercise, males = 24.469 ± 3.911 mmol/kg, females = 18.353 ± 4.818 mmol/kg, *p* = 0.035) had significant sex differences. Males had significantly greater PCr/Pi, but less PCr/ATP than females during exercise, suggesting males had higher energy transfer efficiency than females. At the post-exercise status, the recovery of PCr did not show sex difference.

**Conclusions.** Our in-house force control and gauge system quantitatively applied force during the exercise for ^31^P-MRS experiments, and a sex difference of higher energy transfer efficiency and WE was detected in males with mild loaded exercising quadriceps. This noninvasive technology allows us to further study and understand the sex difference of high energy phosphate metabolism in the future.

## Introduction

Muscle is the only apparatus that can transform energy into force and movement in the human body. Generally, males display greater strength and muscle capacity than females while performing a task. Former histochemical and biochemical studies demonstrated that there were many differences in both fiber type composition and contractile speed of skeletal muscle between males and females (Rhee et al., 2015; Simoneau et al., 1985), with males having a significantly higher overall capacity for aerobic oxidation and for anaerobic glycolysis (Green, Fraser & Ranney, 1984). Although, a muscle needle biopsy is often regarded as the gold standard in evaluating muscle, the small tissue sample may not be representative for the entire muscle and is perhaps just a single snapshot of resting muscle (Sargeant & De Haan, 2006). Due to the technical challenges and their invasiveness, biopsies are usually not repeatable in dynamic studies.

The ^31^P magnetic resonance spectroscopy (^31^P-MRS) methods concentrate on high-energy phosphate metabolism in skeletal muscle to better understand the physiology of mitochondrial ATP synthesis (Kemp et al., 2015). In contrast to muscle biopsies, ^31^P-MRS is noninvasive, continuous and repeatable (Layec et al., 2009). It allows tracking of real time changes in the relative concentrations of metabolites that are involved in high- energy phosphate metabolism such as adenosine triphosphate (ATP), phosphocreatine (PCr), inorganic phosphate (Pi) as well as changes in muscle pH. The oxidative recovery rate constant and oxidative capacity can be further calculated. It makes more sense to examine high energy phosphate metabolism during exercise. So far, this technique has been widely used to study energy metabolism and oxidative capacity of muscle in several skeletal muscle and endocrine disorders (Rana et al., 2014; Sun et al., 2014; Rana et al., 2012; Wu et al., 2012; Befroy et al., 2012).

In former studies, many histological examinations showed the difference of skeletal muscle (vastus lateralis muscle and tibialis anterior muscle) between males and females such as muscle cross-sectional area and volume, fibre type composition and maximal enzyme activities, etc. (Simoneau et al., 1985; Green, Fraser & Ranney, 1984; Jaworowski et al., 2002). However, few studies of sex differences in phosphate metabolism of lower limb skeleton muscle by ^31^P-MRS have been reported. A ^31^P-MRS study of gastrocnemius in healthy Indian men and women exhibited no sex differences in phosphate metabolism, oxidative recovery rate constant and oxidative capacity (Rana et al., 2008). Another ^31^P-MRS study of quadriceps from Germany shows that pH was the only one of the evaluated spectroscopic parameters that showed a sex dependence (Schunk et al., 1999). Both of the above ^31^P-MRS studies used a 1.5T MR scanner with a shorter repetition time (TR) of 1,500 ms, which potentially may generate unstable spectral data. The German study also used an un-individauted sandbag with constant weight as an exercise load. This problem was addressed by using an in-house fabricated wooden ergometer from the study in India. In the present study, ^31^P-MRS was performed in 3.0T MRI scanner using a longer TR of 5,000 ms and the exercise load was controlled by a self-designed force control and gauge system.

## Material and Methods

### Ethical approval

This study was approved by the ethical committee of the Affiliated Drum Tower Hospital of Nanjing University Medical School (Number AF/SQ-03/02.6). The study complied with the Declaration of Helsinki. Oral and informed consent was obtained from all volunteers.

**Figure 1:**
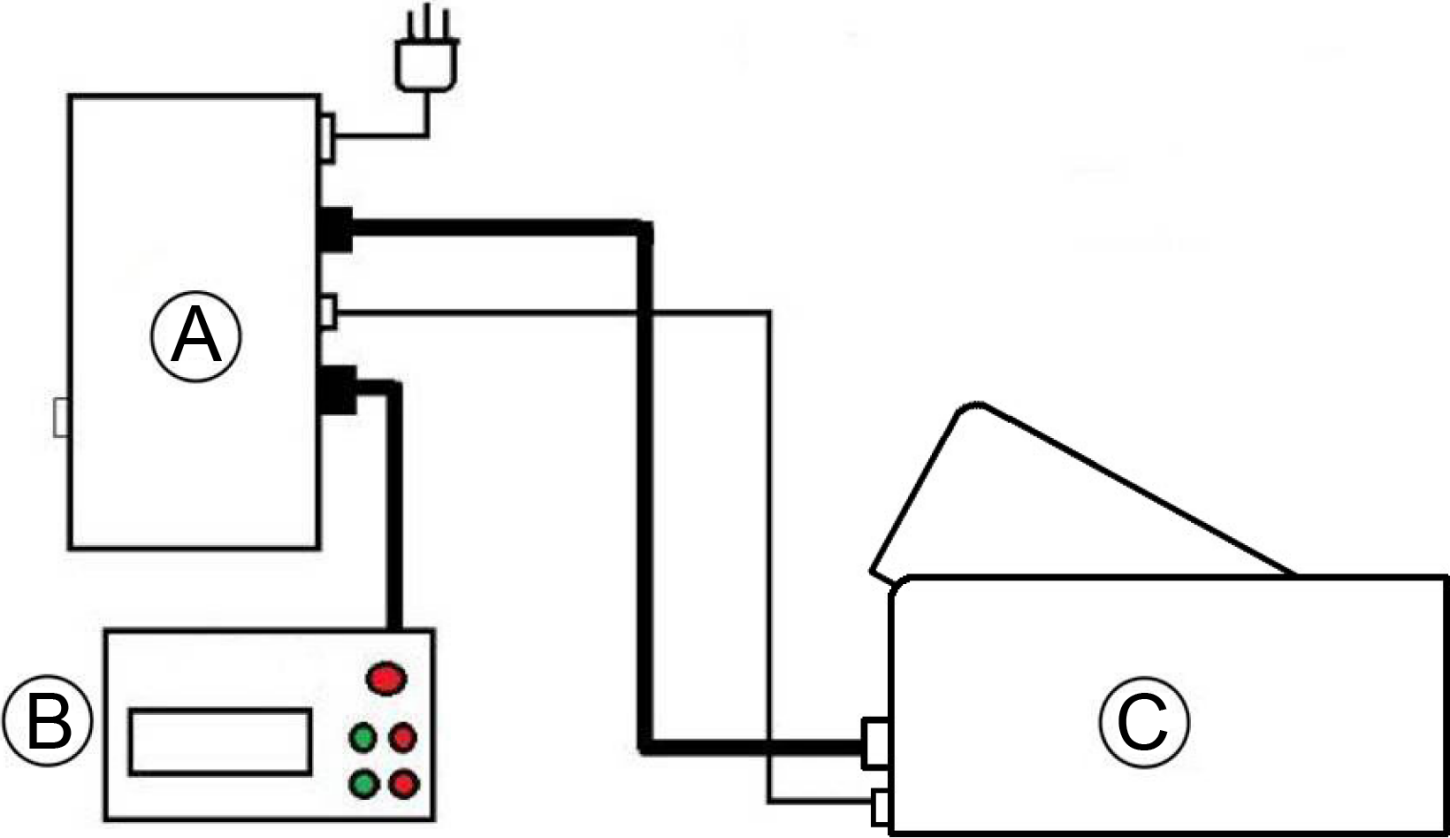
The custom built MR compatible quantitative force control system. (A) is a computer control system, (B) is a digital panel with “feedback-mechanism” and (C) is a knee-extending excises pedal made with plastic.

### Subject

This study was approved by the ethical committee of the Affiliated Drum Tower Hospital of Nanjing University Medical School. A total of 17 healthy volunteers (10 male and 7 female) with similar physical activity habits, males aged 21–25 yr (mean 23.30 ± 1.25 yr) and females aged 22–24 yr (mean 23.57 ± 0.79 yr), performed knee-extending excises while lying prone in Philips 3T MRI scanner (Achieva 3.0T TX, Eindhoven, Netherlands) using a force control and gauge system (Fig. 1). All subjects had no musculoskeletal, cardiovascular or endocrine diseases and were not undergoing any treatment for any other diseases. For all subjects, health status was assessed based on medical records and physical examination.

### Force control and gauge system

The module with a knee-extending pedal (400 mm × 215 mm × 195 mm) was specially designed in-house to stress-test the quadriceps (Fig. 1). The maximum standard torque of this equipment is 80 Nm.

### ^31^P-MRS acquisition

A transmitter/receiver surface coil (dimension 14 cm × 14 cm, Philips, Netherlands) was used to obtain ^31^P-MRS. The coil was firmly placed under the middle of the quadriceps. ^31^P-MRS sequence was obtained using the following parameters: TR = 5,000 ms, TE = 0.1 ms, NSA 8, VOI = 30 mm × 30 mm × 120 mm, acquisition time = 50 s. The ^31^P-MRS sequence was acquired at three phases: Phase 1 (pre-exercise): the resting stage; Phase 2 (during-exercise): dynamic measurements were taken of the loaded quadriceps with a 25% maximum voluntary contraction (MVC) (Skurvydas et al., 2008), this sequence was repeated six times, lasting 5 min; Phase 3 (post-exercise): recovery measurements were taken with the muscle at rest, sequence repeated six times, lasting for 5 min. The initial MVC for each subject’s quadriceps muscles was determined before ^31^P-MRS acquisition, measured by maximum load difference between maximum force during exercise and at rest. The subject pushed the load by extension of the lower leg once every 5 s over a 5-minute interval.

### Metabolite measurements and PCr recovery kinetics

^31^P-MRS measurements of inorganic phosphate (Pi), phosphocreatine (PCr) and adenosine triphosphate (ATP) were acquired from quadriceps while subjects were in a state of pre-, during- and post-exercise. Resonance areas of Pi, PCr, and ATP were corrected for saturation effects, and relative concentrations of them were determined from the corrected resonance areas as detailed in previous reports (Park et al., 2000). The ATP value is equal to the sum of three different subtypes of ATP (*α*-ATP, *β*-ATP and *γ*-ATP). The absolute PCr and Pi concentrations were calculated using PCr/*β*-ATP, PCr/Pi ratios, assigning a concentration of 5.5 mmol kg^−1^ muscle wet weight to the average resting value of the *β*-ATP peak. The PCr, Pi, PCr/Pi, PCr/ATP, pH, work/energy cost ratio (WE), *k*_PCr_ and oxidative capacity of males and females were calculated respectively. The pH value was calculated according to chemical shifts of Pi and PCr by using the following formula: pH = 6.75 + log(Δ − 3.27)∕(5.69 − Δ), where Δ is the chemical distance between Pi and PCr [8]. The exercise load per body weight (L/W, exercise load/bodyweight) was calculated as the true load situation to compare between males and females. The WE of muscles was calculated as the load (kg) divided by the Pi:PCr ratio (Park et al., 2000). The linear model of oxidative phosphorylation was used for studying recovery kinetics. The *k*_PCr_ is an oxidative recovery rate constant, which was calculated according to the following formula: [PCr]_*t*_ = [PCr]_0_ + Δ[PCr](1 − exp^(−*t*∕*τ*)^), where [PCr]_0_ is the level at the beginning of recovery, Δ[PCr] = [PCr]_rest_ − [PCr]_0_ and *k*_PCr_ = 1∕*τ*, where *τ* = time constant. Oxidative capacity was estimated for each subject by the product of *k*_PCr_ and [PCr]_rest_ (Rana et al., 2008).

### Statistical methods

Measurement data was presented as mean ± standard deviation (SD). Statistical Package for Social Sciences (SPSS) version 20.0 software (SPSS Inc., IL, USA) was used for statistical analysis. The differences in data between males and females were evaluated by independent-samples *T* test (two-tailed). Paired-samples *T* test was used to calculate the differences of metabolites between pre-exercise and post-exercise. Each phosphate metabolite ratio among three phases was compared using one-way analysis of variance (ANOVA), multiple comparison using LED test (equal variances) and Tamhane test (unequal variances) based on the results of a homogeneity test of variances. *P*-value < 0.05 was considered statistically significant.

## Results

### General information

17 volunteers underwent the study. Males: *N* = 10, age = 23.30 ± 1.25 years; females: *N* = 7, age = 23.57 ± 0.79 years. All volunteers carried out the exercise within the magnet according to the protocols and did not come across any problems. Although average height (males = 1.74 ± 0.06 m, females = 1.63 ± 0.06 m, *p* = 0.002), weight (males = 66.10 ± 10.15 kg, females = 53.14 ± 5.18 kg, *p* = 0.008) and exercise load (3.03 ± 0.78 kg vs. 2.18 ± 0.73 kg, *p* = 0.040) of male were taller, heavier and greater than female, there were no statistically significant differences in age, BMI and L/W between them. In this study, males had significantly greater WE (16.33 ± 6.46 vs. 7.82 ± 2.16, *p* = 0.002) than females. The detailed data is presented in Table 1 and Supplemental Dataset.

**Table 1:** The general information and work efficiency of male and female groups.

	Age (yr)	Height (m)	Weight (Kg)	BMI	Load (kg)	L/W	WE
F	23.57 ± 0.79	1.63 ± 0.06^a^	53.14 ± 5.18^a^	20.01 ± 1.17	2.18 ± 0.73^a^	0.05 ± 0.01	7.82 ± 2.16^a^
M	23.30 ± 1.25	1.74 ± 0.06	66.10 ± 10.15	21.78 ± 2.20	3.03 ± 0.78	0.04 ± 0.01	16.33 ± 6.46
*t*	0.505	−3.699	−3.088	−1.933	−2.255	0.950	−3.866
*P*	0.621	0.002	0.008	0.072	0.040	0.357	0.002

**Notes.**

Data is shown as mean ± SD.

BMI body mass index L/W exercise load per body weight (Load/Weight) WE work/energy cost ratio F female M male

a Significant difference between males and females. *P* < 0.05 means statistically significant difference.

### Dynamic ^31^P-MRS results

**Figure 2:**
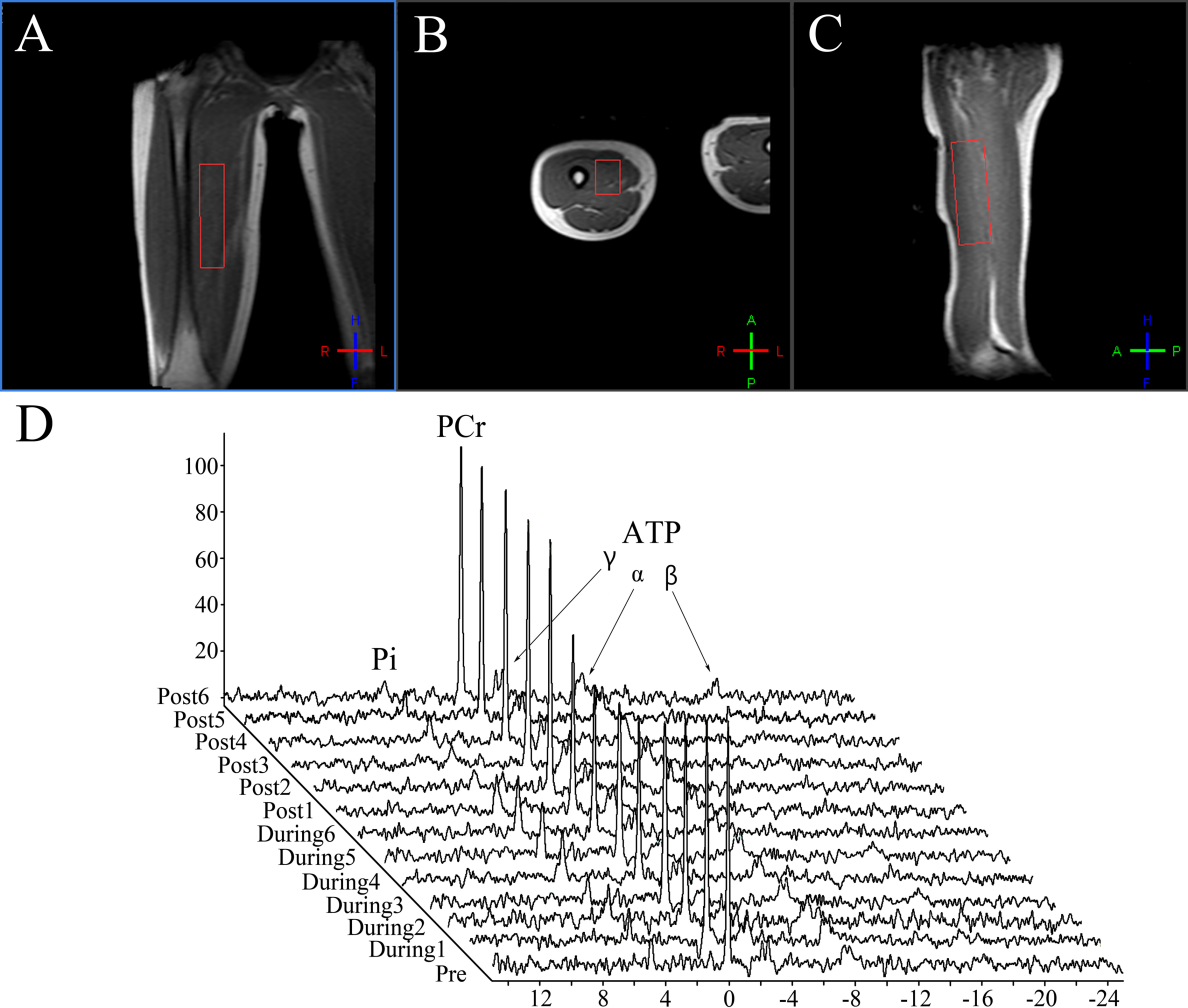
The ^31^P magnetic resonance spectroscopy obtained from a representative subject. (A–C) show a representative anatomical location image with the ROI volume box. (D) shows a representative ^31^P magnetic resonance spectroscopy from one subject.

**Figure 3:**
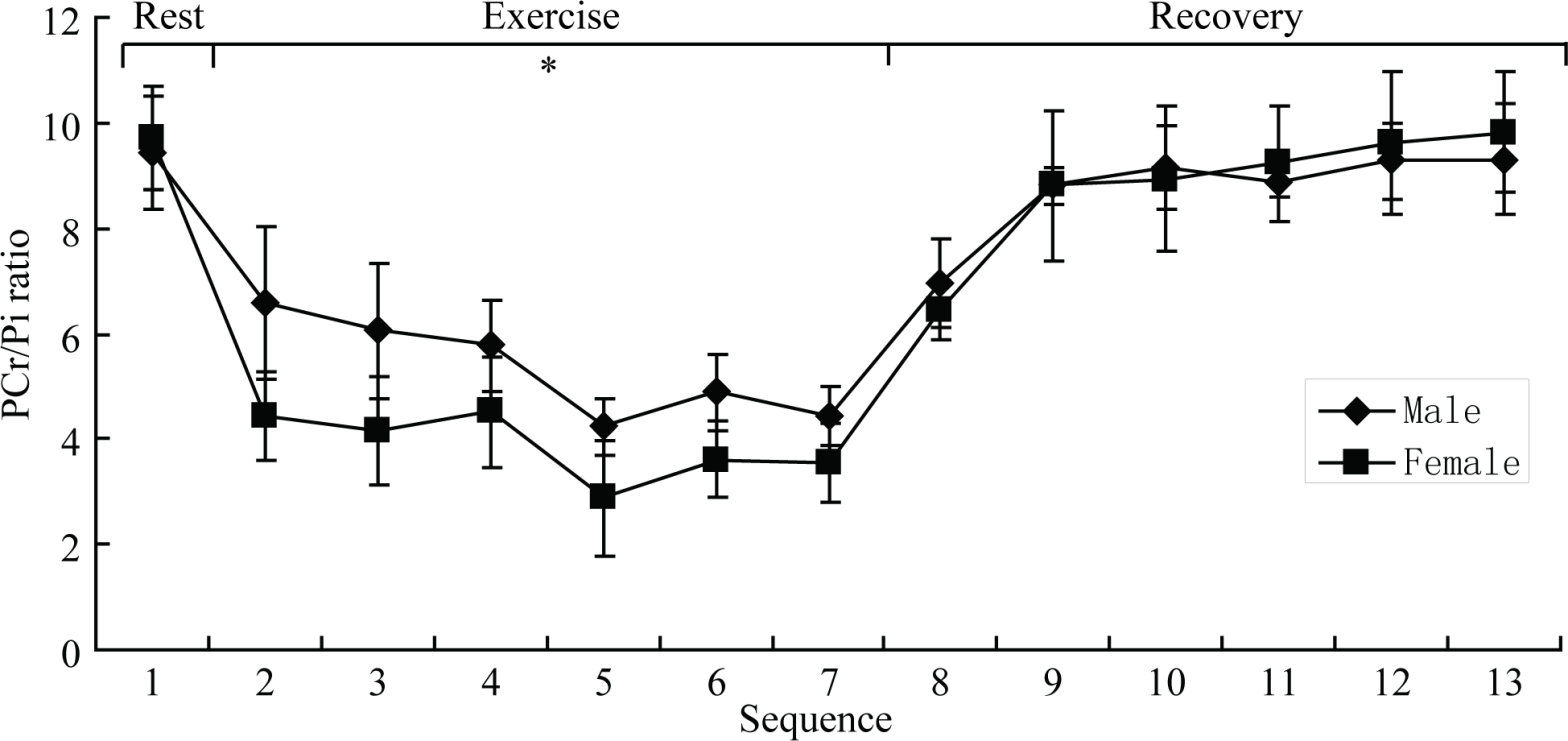
Group mean (±SD) for the time course of phosphocreatine/inorganic phosphate ratio. ^∗^ Denotes statistically significant difference between males and females. *P* < 0.05 means statistically significant difference.

Figure 2 shows a typical ^31^P-MRS spectra from the quadriceps of one volunteer. It displays five major peaks representing Pi, PCr, *α*-ATP, *β*-ATP and *γ*-ATP. The metabolite concentration, metabolite ratios and other parameters are shown in Table 2 and Supplemental Dataset. Among PCr, Pi, PCr/Pi, PCr/ATP, pH, kPCr and oxidative capacity at different exercise status, only PCr/Pi (during-exercise, males = 5.630 ± 1.647, females = 4.014 ± 1.298, *p* = 0.047), PCr/ATP (during-exercise, males = 1.273 ± 0.219, females = 1.523 ± 0.167, *p* = 0.025), and ATP (post-exercise, males = 24.469 ± 3.911 mmol/kg, females = 18.353 ± 4.818 mmol/kg, *p* = 0.035) had significant sex differences. Males had significantly greater PCr/Pi, but less PCr/ATP than females during exercise, suggesting males had higher energy transfer efficiency than females. The PCr (females, pre-exercise = 47.948 ± 9.308 mmol/kg, post-exercise = 32.969 ± 6.387 mmol/kg, *p* = 0.018) and ATP (females, pre-exercise = 27.967 ± 4.521 mmol/kg, post-exercise = 18.353 ± 4.818 mmol/kg, *p* = 0.012) concentration of two groups decreased post-exercise, nevertheless, only that of females had significant differences compared to pre-exercise. The time-course of the changes in PCr/Pi ratio from pre-exercise to post-exercise for both male and female groups is displayed in Fig. 3. There was a sharp decline in PCr/Pi ratio of both groups during-exercise. Table 2 shows the PCr/Pi ratio had significant differences among different phases for the female group, however the significant difference only existed between pre-exercise and other two phases for the male group. Post-exercise, the pH level (males, pre-exercise = 7.012 ± 0.019, post-exercise = 6.983 ± 0.026, *p* = 0.012) (females, pre-exercise = 6.995 ± 0.024, post-exercise = 6.962 ± 0.018, *p* = 0.045) significantly reduced compared to that of pre-exercise both in male and female groups.

**Table 2:** The detail metabolite levels of male and female groups determined by ^31^P magnetic resonance spectroscopy and statistical result of them.

*Parameters*		*Male*	*Female*	P_4_
	Pre-exercise	9.437 ± 2.175^a^	9.699 ± 1.944^a^	0.806
PCr/Pi	During-exercise	5.630 ± 1.647^d^	4.014 ± 1.298^c^	0.047
	Post-exercise	6.941 ± 1.676^b^	6.432 ± 1.117^b^	0.527
		*F* = 10.346, *P* = 0.001	*F* = 24.808, *P* = 0.000	
		*P*_1_ = 0.000, *P*_2_ = 0.008, *P*_3_ = 0.134	*P*_1_ = 0.000, *P*_2_ = 0.001, *P*_3_ = 0.011	
	Pre-exercise	1.655 ± 0.354	1.888 ± 0.340	0.354
PCr/ATP	During-exercise	1.273 ± 0.219^d^	1.523 ± 0.167	0.025
	Post-exercise	1.725 ± 0.762	1.896 ± 0.484	0.608
		*F* = 1.674, *P* = 0.209	*F* = 2.519, *P* = 0.109	
		*P*_1_ = 0.179, *P*_2_ = 0.800, *P*_3_ = 0.097	*P*_1_ = 0.071, *P*_2_ = 0.966, *P*_3_ = 0.065	
	Pre-exercise	46.051 ± 9.782	47.948 ± 9.308	0.874
PCr (mmol/kg)	Post-exercise	34.024 ± 7.632	32.969 ± 6.387^b^	0.850
	*P*_2_	0.253	0.018	
	Pre-exercise	5.124 ± 1.035	5.112 ± 1.004	0.989
Pi (mmol/kg)	Post-exercise	5.006 ± 1.305	5.279 ± 1.291	0.817
	*P*_2_	0.914	0.825	
	Pre-exercise	28.775 ± 6.983	27.967 ± 4.521	0.823
ATP (mmol/kg)	Post-exercise	24.469 ± 3.911^d^	18.353 ± 4.818^b^	0.035
	*P*_2_	0.173	0.012	
	Pre-exercise	7.012 ± 0.019	6.995 ± 0.024	0.264
pH	Post-exercise	6.983 ± 0.026^b^	6.962 ± 0.018^b^	0.110
	*P*_2_	0.012	0.045	
*k*_PCr_(s^−1^)		0.012 ± 0.003	0.011 ± 0.003	0.929
Oxidative capacity (mmol/kg s^−1^)		0.536 ± 0.130	0.549 ± 0.127	0.892

**Notes.**

During-exercise denotes the average level of all total six ^31^P-MRS spectra of during-exercise. Post-exercise denotes the first repeat ^31^P-MRS spectrum among total six spectra of post-exercise. *P* Compares pre-, during- and post-exercise. *P*_1_ Compares pre- to during-exercise. *P*_2_ Compares pre- to end-exercise. *P*_3_ Compares during- to post-exercise. *P*_4_ Compares males with females.

a Significant difference between pre- and during- exercise.

b Significant difference between pre- and post-exercise.

c Significant difference between during- and post-exercise.

d Significant difference between males and females. *P* < 0.05 means statistically significant difference.

### Oxidative phosphorylation parameters

**Figure 4:**
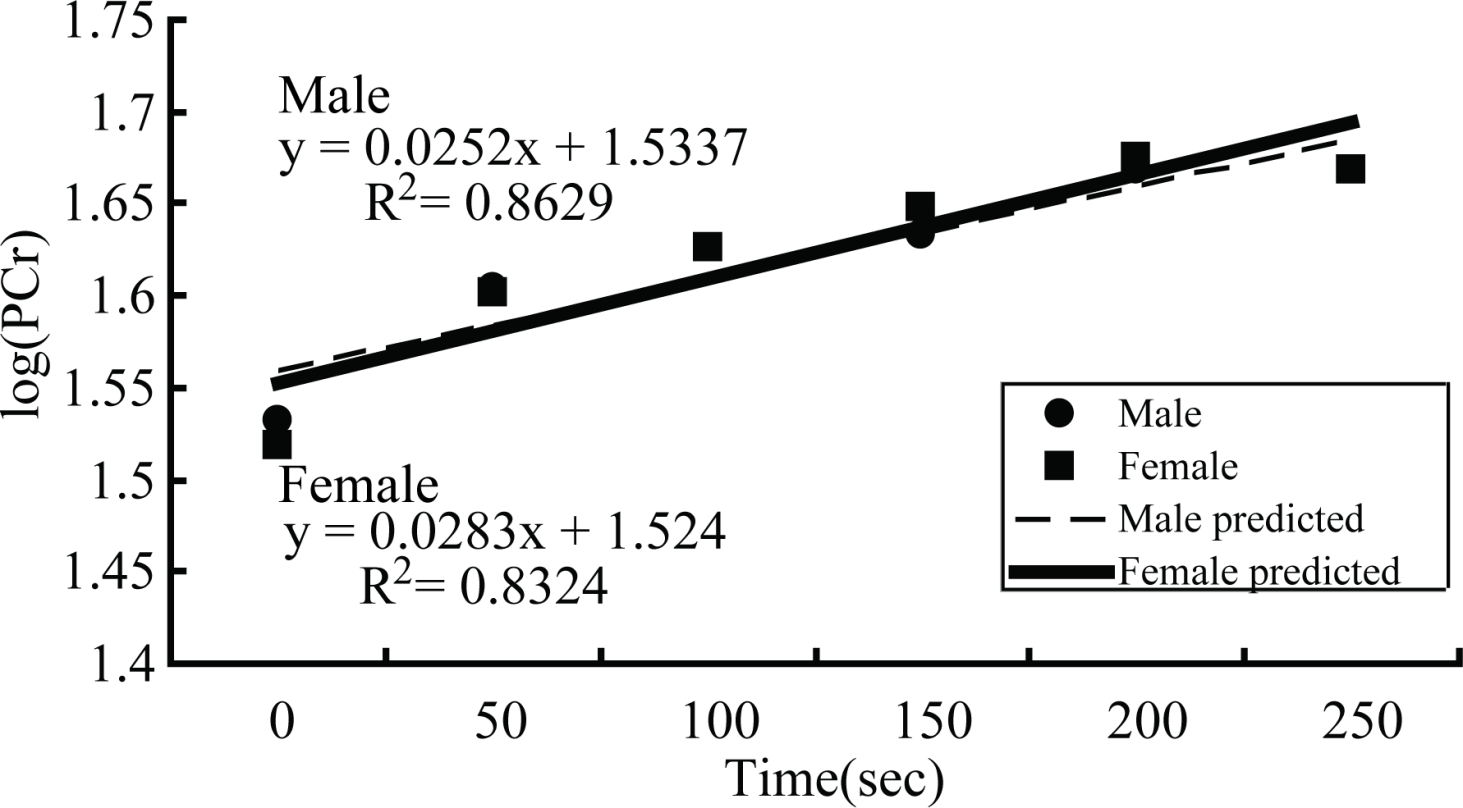
The recovery of phosphocreatine. Recovery line of logarithmic PCr predicted by a linear fit model for male and female groups.

At the post-exercise status, the recovery of PCr did not show sex difference. The recovery log(PCr) could adopt a linear fit model to calculate values for both groups (Fig. 4). The mean values of *k*_PCr_ and oxidative capacity of male and female groups were shown in Table 2. Although the *k*_PCr_ of males was slight higher and oxidative capacity was slightly lower than that of females, there were no difference of *k*_PCr_ and oxidative capacity between male and female groups.

## Discussion

We applied a dynamic ^31^P-MRS to study dynamic changes in phosphate metabolite levels in exercising quadriceps, in order to study the differences between males and females under various metabolic states. Our results indicate that multi-dynamic ^31^P-MRS could effectively monitor the dynamic change of PCr, Pi and ATP.

In our study, the PCr/Pi ratio significantly decreased in during-exercise compared to pre-exercise in both males and females which closely matches with previously published research (Sun et al., 2014; Wu et al., 2012; Rana et al., 2008; Schmitz et al., 2012; Wu et al., 2011; Meyerspeer et al., 2011) (Table 2 and Fig. 3). Although PCr/ATP also had a downtrend in during-exercise, it was not statistically significant. It is mainly because, in order to provide energy for muscular contraction, the high-energy molecule ATP splits into adenosine diphosphate (ADP), Pi, as well as [H^+^] ions by the ATPase enzyme. In order to re-phosphorylate ADP to ATP, a phosphate group of the PCr is transferred to ADP by the creatine kinase reaction. PCr acts as an intermediate energy buffer and ‘shuttles’ energy-rich phosphates from the mitochondria to the myofibrils (Gouspillou et al., 2014). In brief, PCr decreases as a result of PCr hydrolysis which maintains ATP homeostasis, crucial energy source readily used for muscle contraction (Wu et al., 2011), with concomitant rise in Pi. But, to our knowledge, we are the first to report that PCr/Pi of males was significantly higher than that of females during-exercise, while PCr/ATP had the opposite trend. Considering that PCr/Pi is widely regarded as a sensitive marker of the muscle’s energy reserve and oxidative capacity, it also represents the transfer efficiency of intracellular high-energy phosphate bonds (Wu et al., 2012; Jones et al., 2008). It may be inferred that there is a higher oxidative capacity and/or energy transfer efficiency in male. According to our data, we found calculated *k*_PCr_ and oxidative capacity to have no sex statistical difference. So, we concluded this sex difference is conceivably attributed to a higher energy transfer efficiency in males.

We found that PCr and ATP concentrations decreased both in males and females post-exercise, which represents PCr and ATP consumption to maintain the energy expenditure needed for exercise, but interestingly, there was only statistical differences in females. Besides, the PCr/Pi of females during-exercise was significantly lower than post-exercise, which was not found in the male group. There were no significant differences of PCr and Pi concentration between males and females post-exercise, however, the ATP concentration of females was less than that of males. Based on our results, there was sufficient evidence to conclude that females may have a greater consumption than males in exercise, which is consistent with our results that the WE of females exceeded that of males. This difference may be related to the difference in fiber type composition and contractile speed of skeletal muscle between males and females (Simoneau et al., 1985).

In our study, the PCr recovery curve could be predicted by a monoexponential fit model for male and female groups (Fig. 4A), a technique demonstrated in earlier published articles (Wu et al., 2012; Rana et al., 2008; Wu et al., 2011). Furthermore, we used a logarithmic algorithm to change PCr values to log(PCr), as a consequence, the recovery log(PCr) could adopt a linear fit model to calculate values of both groups (Fig. 4B). Our study did not demonstrate any significant differences in pH values at rest (pre-exercise) between males and females, which agrees with earlier investigations (Rana et al., 2008; Conley, Jubrias & Esselman, 2000), along with the pH values of the quadriceps post-exercise. The pH decreased post-exercise on the order of some other related ^31^P-MRS research (Sun et al., 2014; Park et al., 2000; Jones et al., 2008), which is in line with our present study. As mentioned before, the ATP splits into ADP and Pi as well as [H^+^] ions as a result of the ATPase enzyme function, but in order to re-phosphorylate ADP to ATP, a phosphate group of the PCr must be transferred to ADP by the creatine kinase reaction. PCr acts as an intermediate energy buffer and ‘shuttles’ energy-rich phosphates from the mitochondria to the myofibrils (Gouspillou et al., 2014). Under this buffering, [H^+^] ion metabolism is balanced, and the pH remains unchanged at the onset of muscular work. The concentration of [H^+^] ions increased in line with the intensity of exercise continuing on until broken by the buffering of PCr, performed with a decreased pH value at the end of exercise.

In our study, the exercise load between females and males was different this has potential effect on the sex differences of PCr/Pi, PCr/ATP and ATP. Because we adopt the load program according to individual 25% MVC controlled by our force control and gauge system, which leads to the different exercise workload between males and females. However, generally, males display greater strength (load) and muscle capacity (weight) than females while performing a task. Therefore, we calculated L/W as the true load situation to compare between males and females. The result demonstrated there was no difference in load per bodyweight between males and females. Therefore, we think the individual load maybe more significant than the same exercise load to every subject with different body weight for detecting individual phosphate metabolisms and oxidative capacity.

In addition, 25% MVC should be considered as mild exercise condition. We need to find out if the conclusion from this study (25% MVC) could be generalized to other exercise situations (such as 50% or 75% MVC) or be valid for the same exercise workload to both males and females in our future study. We did try the same experience with 50% or 75% MVC in our preliminary study. However, 50% or 75% MVC is too much load to maintain the regular exercise in whole 5 min for most of subjects, especially for females. Maybe this question can be ruled out by applying different muscle loading to males only, and to evaluate if one could find differences of PCr/Pi, PCr/ATP and ATP between small muscle loading and large muscle loading group in males. Although most of subjects in this study couldn’t tolerate large muscle loading, maybe we can recruit other appropriate volunteers (trained before) to fulfill this different load experience in the next work.

### Limitations

Our study had several limitations. Firstly, the pedal of force control and gauge system is not adjustable for different individual length of crus, which potentially leads to deviation of the load calculation. However, to reduce the limitation as possible, we trained every volunteer strictly and matted the cotton cushion with variable thickness to control the same location of toes to the best of our ability. Secondly, the exercising muscles potentially generate a motion artifact during MRI acquisition, which was controlled in this study by firmly placing the ^31^P-MRS surface coil under the quadriceps and hold it tightly in place with cotton slivers to reduce the relative movement, in turn reducing the motion artifact and increasing the signal to noise ratio. Another limiting factor in this study was that the sample size was relatively small, and so the reliability and stability of the ^31^P-MRS approach applied on the quadriceps requires further confirmation with more subjects in the next following study.

## Conclusions

Our in-house force control and gauge system quantitatively applied force during the exercise for ^31^P-MRS experiments, and a sex difference of higher energy transfer efficiency and WE was detected in males with mild loaded exercising quadriceps. This noninvasive technology allows us to further study and understand the sex difference of high energy phosphate metabolism in the future.

##  Supplemental Information

10.7717/peerj.2259/supp-1 Data S1 Raw ^31^P magnetic resonance spectroscopy images and datasource from male group Each phase of ^31^P magnetic resonance spectroscopy images and datasource from each volunteer in male group.

10.7717/peerj.2259/supp-2 Data S2 Raw ^31^P magnetic resonance spectroscopy images and datasource from female group Each phase of ^31^P magnetic resonance spectroscopy images and datasource from each volunteer in female group.

10.7717/peerj.2259/supp-3 Data S3 The detail raw data from raw ^31^P magnetic resonance spectroscopy images and datasource

## Additional Information and Declarations

### Data Availability

The following information was supplied regarding data availability: The raw data has been supplied as a Supplemental Dataset.

## References

[ref-1] Befroy DE, Rothman DL, Petersen KF, Shulman GI (2012). (3)(1)P-magnetization transfer magnetic resonance spectroscopy measurements of *in vivo* metabolism. Diabetes.

[ref-2] Conley KE, Jubrias SA, Esselman PC (2000). Oxidative capacity and ageing in human muscle. Journal of Physiology.

[ref-3] Gouspillou G, Bourdel-Marchasson I, Rouland R, Calmettes G, Biran M, Deschodt-Arsac V, Miraux S, Thiaudiere E, Pasdois P, Detaille D, Franconi JM, Babot M, Trézéguet V, Arsac L, Diolez P (2014). Mitochondrial energetics is impaired *in vivo* in aged skeletal muscle. Aging Cell.

[ref-4] Green HJ, Fraser IG, Ranney DA (1984). Male and female differences in enzyme activities of energy metabolism in vastus lateralis muscle. Journal of the Neurological Sciences.

[ref-5] Jaworowski A, Porter MM, Holmback AM, Downham D, Lexell J (2002). Enzyme activities in the tibialis anterior muscle of young moderately active men and women: relationship with body composition, muscle cross-sectional area and fibre type composition. Acta Physiologica Scandinavica.

[ref-6] Jones AM, Wilkerson DP, DiMenna F, Fulford J, Poole DC (2008). Muscle metabolic responses to exercise above and below the “critical power” assessed using 31P-MRS. American Journal of Physiology. Regulatory, Integrative and Comparative Physiology.

[ref-7] Kemp GJ, Ahmad RE, Nicolay K, Prompers JJ (2015). Quantification of skeletal muscle mitochondrial function by P magnetic resonance spectroscopy techniques: a quantitative review. Acta Physiologica.

[ref-8] Layec G, Bringard A, Le Fur Y, Vilmen C, Micallef JP, Perrey S, Cozzone PJ, Bendahan D (2009). Reproducibility assessment of metabolic variables characterizing muscle energetics *in vivo*: a 31P-MRS study. Magnetic Resonance in Medicine.

[ref-9] Meyerspeer M, Scheenen T, Schmid AI, Mandl T, Unger E, Moser E (2011). Semi-LASER localized dynamic 31P magnetic resonance spectroscopy in exercising muscle at ultra-high magnetic field. Magnetic Resonance in Medicine.

[ref-10] Park JH, Niermann KJ, Ryder NM, Nelson AE, Das A, Lawton AR, Hernanz-Schulman M, Olsen NJ (2000). Muscle abnormalities in juvenile dermatomyositis patients: P-31 magnetic resonance spectroscopy studies. Arthtitis and Rheumatism.

[ref-11] Rana P, Marwaha RK, Kumar P, Narang A, Devi MM, Tripathi RP, Khushu S (2014). Effect of vitamin D supplementation on muscle energy phospho-metabolites: a P magnetic resonance spectroscopy-based pilot study. Endocrine Research.

[ref-12] Rana P, Sripathy G, Varshney A, Kumar P, Devi MM, Marwaha RK, Tripathi RP, Khushu S (2012). Phosphorous magnetic resonance spectroscopy-based skeletal muscle bioenergetic studies in subclinical hypothyroidism. Journal of Endocrinological Investigation.

[ref-13] Rana P, Varshney A, Devi MM, Kumar P, Khushu S (2008). Non-invasive assessment of oxidative capacity in young Indian men and women: a 31P magnetic resonance spectroscopy study. Indian Journal of Biochemistry and Biophysics.

[ref-14] Rhee KY, Kim TY, Oh IS, Lee SJ, Ledowski T (2015). Effect of muscle relaxation on the oxygenation of human skeletal muscle: a prospective *in-vivo* experiment using an isolated forearm technique. Korean Journal of Anesthesiology.

[ref-15] Sargeant AJ, De Haan A (2006). Human muscle fatigue: the significance of muscle fibre type variability studied using a micro-dissection approach. Journal of Physiology and Pharmacology.

[ref-16] Schmitz JP, Jeneson JA, Van Oorschot JW, Prompers JJ, Nicolay K, Hilbers PA, Van Riel NA (2012). Prediction of muscle energy states at low metabolic rates requires feedback control of mitochondrial respiratory chain activity by inorganic phosphate. PLoS ONE.

[ref-17] Schunk K, Pitton M, Duber C, Kersjes W, Schadmand-Fischer S, Thelen M (1999). Dynamic phosphorus-31 magnetic resonance spectroscopy of the quadriceps muscle: effects of age and sex on spectroscopic results. Investigative Radiology.

[ref-18] Simoneau JA, Lortie G, Boulay MR, Thibault MC, Theriault G, Bouchard C (1985). Skeletal muscle histochemical and biochemical characteristics in sedentary male and female subjects. Canadian Journal of Physiology and Pharmacology.

[ref-19] Skurvydas A, Masiulis N, Stanislovaitis A, Kamandulis S (2008). Bi-modal recovery of quadriceps femoris muscle function after sustained maximum voluntary contraction at different muscle length. Medicina.

[ref-20] Sun Y, Pan S, Chen Z, Zhao H, Ma Y, Zheng L, Li Q, Deng C, Fu X, Lu Z, Guo Q (2014). Changes in energy metabolism in the quadriceps femoris after a single bout of acute exhaustive swimming in rats: a (3)(1)P-magnetic resonance spectroscopy study. Chinese Medical Journal.

[ref-21] Wu JS, Buettner C, Smithline H, Ngo LH, Greenman RL (2011). Evaluation of skeletal muscle during calf exercise by 31-phosphorus magnetic resonance spectroscopy in patients on statin medications. Muscle and Nerve.

[ref-22] Wu FY, Tu HJ, Qin B, Chen T, Xu HF, Qi J, Wang DH (2012). Value of dynamic (3)(1)P magnetic resonance spectroscopy technique in *in vivo* assessment of the skeletal muscle mitochondrial function in type 2 diabetes. Chinese Medical Journal.

